# Jambu Flower Extract (*Acmella oleracea*) Increases the Antioxidant Potential of Beer with a Reduced Alcohol Content

**DOI:** 10.3390/plants12081581

**Published:** 2023-04-07

**Authors:** Suelem Paixão da Silva, José Augusto Lacerda Fernandes, Alberdan Silva Santos, Nelson Rosa Ferreira

**Affiliations:** 1 Graduate Program in Food Science and Technology, Federal University of Pará, Belém 66077-000, PA, Brazil; 2 Faculty of Administration, Federal University of Pará, Belém 66075-110, PA, Brazil; 3 Faculty of Chemistry, Institute of Exact and Natural Sciences, Federal University of Pará, Belém 66075-110, PA, Brazil; 4 Faculty of Food Engineering, Technology Institute, Federal University of Pará, Belém 66077-000, PA, Brazil

**Keywords:** craft beer, *Acmella oleracea*, spilanthol, antioxidant activity, reduced alcohol content

## Abstract

Craft beers with different sensory perceptions have received the attention of more demanding consumers. In this sense, the application of plant extracts as brewing adjuncts is being increasingly studied. Allied with these perspectives is the consumption of lower alcoholic beverages, which also represents the desire for a market niche that has been growing gradually. The present work aimed to produce craft lager beer with the addition of plant extract and reduced alcohol content by partial replacement of malt with malt bagasse. The physical-chemical analyzes of the beer produced showed that it was possible to reduce the alcohol content by 40.5% compared to the control sample. In addition, an extract of *Acmella oleracea* (Jambu) obtained by supercritical extraction was added to increase the beer’s antioxidant capacity. The ABTS, DPPH, and ORAC methods evaluated the antioxidant capacity. These assays were performed again after six months of storage. The quantification and identification of the significant substance in the extract (spilanthol) were performed using Gas Chromatography (GC-FID), Thin Layer Chromatography (TLC), and Attenuated Total Reflectance Infrared Spectroscopy (FTIR-ATR). The results showed significant increases in antioxidant activity compared to the sample without extract. This positive aspect opens a perspective for using jambu flower extract as a prominent antioxidant adjunct in beer.

## 1. Introduction

The production and consumption of beer are ancient practices developed by man, and producers are always looking for improvements in the production process and development of new products that can please and meet the expectations of current consumers. Within this context, research is being developed to show possible functional characteristics of beer, indicating that moderate consumption can be part of an adult’s healthy lifestyle [1]. Furthermore, there is consistent evidence in the literature of the antioxidant protection of compounds present in beer [2,3]. In this context, special beers added with certain fruits and herbs rich in bioactive substances with antioxidant capacity are part of this research [4,5,6,7]. Thus, it is an interesting strategy to further increase the content of bioactive substances in beer by adding fruit or plant extracts.

*Acmella oleracea* is a vegetable from the Amazon, widely cultivated and appreciated in the northern region of Brazil. The main constituent of this plant is an n-.alkylamide called spilanthol. This compound is present in other genera, such as *Echinacea* and *Zanthoxylum*, which have proven antioxidant properties [8,9]. Also, the actions as an analgesic [10], anti-inflammatory [11], and bacteriostatic [12] were observed. Like other alkyl amides, spilanthol is an amphiphilic compound with a relatively polar amide and a less polar polyun-saturated alkyl. Thus, it can be extracted from plants using methanol, ethanol, supercritical CO_2_, or hexane [13].

The extraction using supercritical carbon dioxide (CO_2_) presents a good yield of spilanthol, with the advantage of being non-toxic and presenting high volatility, resulting in extracts free of organic solvent and suitable for food and beer [11,14].

An increasingly important point for many consumers is beer’s low alcohol content. Usually, this consumer does not intend to drink the non-alcoholic beverage but accepts the reduction of alcohol concentration. This aspect demonstrates a probable trend in consumption patterns. Thus, new processes for developing low-alcohol beer are constantly being developed, aiming at maintaining sensorial characteristics and physicochemical properties [15,16].

Partial substitution by malt bagasse is a low-cost and little-explored technique for obtaining low-alcohol beers. The malt bagasse comes from beer production itself. However, after the previous mashing consumes most of the extracted sugars, it has a reduced fermentable extract content.

The objective of this study was to produce lager craft beer with reduced alcohol content and jambu extract, as well as to evaluate the influence on the antioxidant capacity of beers. Studies on using jambu extract in beers have not yet been reported.

## 2. Results

### 2.1. A. oleracea Extract Obtained by Supercritical Extraction 

#### 2.1.1. Gas Chromatography with Flame Ionization Detector (GC-FID)

**Figure 1:**
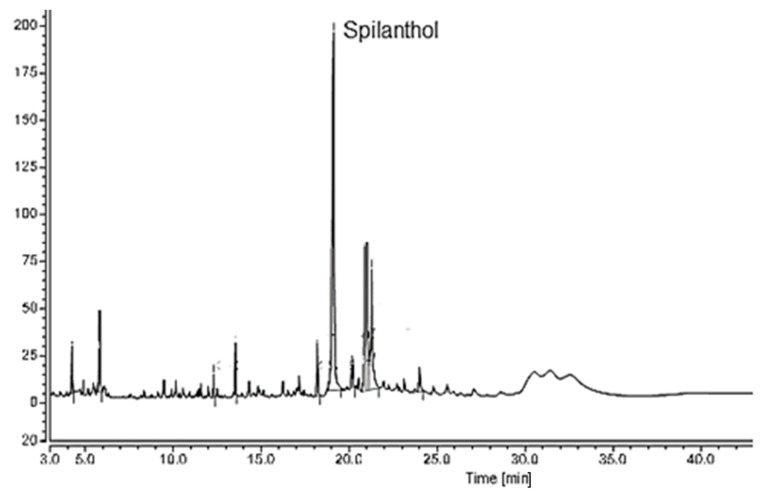
GC-FID chromatogram obtained by the flow of jambu flowers.

The GC-FID analysis confirmed the presence of spilanthol and the relative concentration in the extract obtained by supercritical extraction (S_E_). Figure 1 represents the chromatogram obtained by GC-FID.

The chromatogram shows that spilanthol presented the most prominent peak compared to other components, representing 39.73% of the total extract content, and had a retention time of 19 min. This percentage was superior to those found by Blanco et al. [17], Barbosa et al. [13], and Dias et al. [14]. Among the works found for supercritical extraction of jambu, only Dias et al. [14] used the same temperature and pressure conditions, obtaining a percentage of 34.6%.

The supercritical extraction technique with CO_2_ without adding cossolvating was used due to its applicability in the food industry. Barbosa et al. [13] described carbon dioxide as non-toxic, low cost, and high volatility, providing an extract free of organic solvents. In addition, some authors proved the best efficiency of CO_2_ in the extraction of compounds contained in the *A. oleracea* flower. Dias et al. [11] studied the extracts of leaves, stems, and jambu flowers, obtained by supercritical CO_2_. The authors observed that the flowers have more spilanthol than the other parts of the plant. Thus, using flowers was a strategy for obtaining an extract with greater antioxidant capacity, mainly due to the higher concentration of spilanthol.

#### 2.1.2. FTIR Analysis

**Figure 2:**
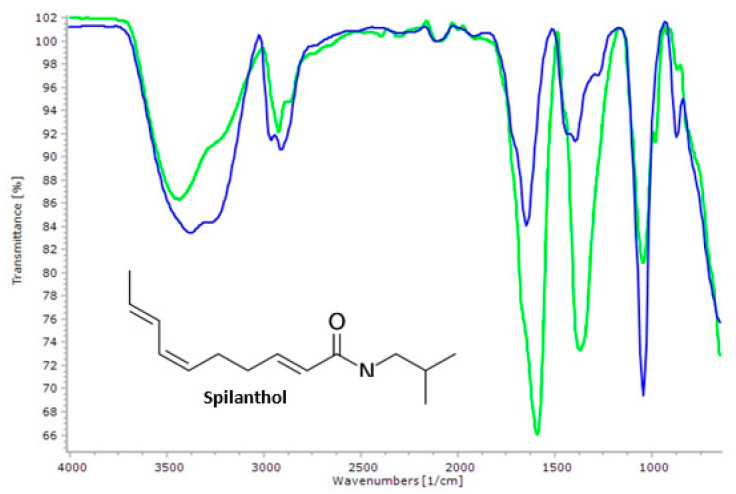
Infrared absorption spectrum in the region from 4000 cm^−1^ to 600 cm^−1^ of supercritical extraction—S_E_ (■) and standard spilanthol—S_S_ (■).

FTIR was used to evaluate and identify the main functional groups present in spilanthol. Figure 2 illustrates infrared spectra from 4000 cm^−1^ to 650 cm^−1^ of the samples S_E_ and standard spilanthol (S_S_). Both correspond to the bands taken from the TLC. A similarity profile is observed in the transmittance bands compared to the respective spectra.

The bands in the region of 3500–3100 cm^−1^ of average intensity correspond to stretching vibrations (ʋ) N-H. A band in 3380 cm^−1^ was observed for S_S_, and another was observed at 3440 cm^−1^. These values indicate the presence of ʋ N-H of amides or amines in the samples. Nakatani and Nagashima [18] reported the same bands in the *A. oleracea* extract. It is important to note that non-replaced (primary) amides CONH_2_ have two bands in the N-H region, while monosubstituted (secondary), CONH, presents only one band. The possibility of the presence of nitrogenous groups is confirmed by the bands at 1645 cm^−1^ to S_S_ and 1605 cm^−1^ for, corresponding to ʋ C=O (amide). Weak angular deformation bands (σ) of amines, located between 1580–1.490 cm^−1^, were not found in the spectrum. Otherwise, it was observed at 1395 cm^−1^ (S_S_) and 1365 cm^−1^ (S_E_) bands corresponding to ʋ C-N; these peaks are characteristic of amides [19]. In the digital printing region (1300–900 cm^−1^), we observed two bands with the same number of waves at 1050 cm^−1^. These bands of strong intensities are related to ʋ C-O, which usually appears between 1300–1000 cm^−1^. Thus, the intense presence of these bands is presumably related to other molecules within the carbonyl group.

### 2.2. Physicochemical Parameters of Beers

The physicochemical characteristics of beers are parameters of classification and quality control of the beverage. Furthermore, any modification in the process and in the raw material can cause changes and result in beers with different characteristics. Thus, the following analyzes were performed: original gravity (OG), final gravity (FG), pH, color (EBC), bitterness (IBU), and alcohol content (ABV%). The results are shown in Table 1.

Original gravity (OG) estimates the amount of sugar present in the wort before fermentation. Thus, the results in Table 1 show that the P_M_ beer had a higher OG than the B_M_ beer since the wort composition of the second beer was changed precisely to reduce the amount of fermentable sugar in the malt. The final gravity (OF) presented results for P_M_ and B_M_ of 1.004 and 1.009, respectively.

The approximate pH values of the beers show that the addition of malt bagasse did not influence this parameter too much. Furthermore, some studies on the characterization of malt bagasse showed that the pH is compatible with what was observed in the wort. Thus, it does not interfere with the acidity level of the wort [20,21,22].

Regarding color, a light malt (Pilsen) was used to give the beer a relatively more neutral aroma, color, and flavor characteristics. Thus, both beers produced were classified as pale beers. This condition was established to evaluate the results inferred by adding jambu extract.

Regarding bitterness, the number of hops was calculated to reach 35 IBU. P_M_ and B_M_ beers obtained approximate values of 31.29 IBU and 29.50 IBU, respectively. These IBU values were above those found by Moura-Nunes et al. [23] who analyzed 29 Brazilian beers from 14 different brands of American lager with a maximum value of 25.5 IBU.

The ABV% results were 4.6% (P_M_) and 2.5% (B_M_). Thus, the partial replacement of malt resulted in a reduction of 45.7% in the alcohol content of the beer. The value of 2.5% was higher than that of Costa [24], with a proportion of 50/50 (malt and malt bagasse). The use of malt bagasse as a technique to reduce alcohol content is still little explored. On the other hand, compared with reducing the alcohol content using special yeasts, a similarity in the results was observed, as in Canonico et al. [25]. They published results of beer with an alcohol content of 2.66 % *v*/*v* using *Torulospora delbrueeckii* yeast. Another similar result was found by Bellut et al. [26], who produced beers with an alcohol content of 2.1% and 2.6% *v*/*v* using *Lachancea fermentati* yeast in low original gravity wort. Thus, the results demonstrate that partial malt replacement can be used to obtain low-alcohol beers. Furthermore, the results in Table 1 also show that adding the A_CE_ did not affect the physicochemical parameters compared to the control. The extract was added only in the bottling process, where many biochemical transformations of the beer have already taken place. All analytical parameters obtained results within the ranges according to the beer formulation.

### 2.3. Total Phenolics Content (TPC)

There are still no reports of published works on the antioxidant activity of adding jambu extract to beers. Therefore, comparisons with other studies were based on beers added with herbs or fruits. The *B_M_* beers were analyzed for antioxidant activities (Aa), and the results of TPC and Aa and their respective replications after six months of storage are also expressed in Table 2.

The TPC analysis showed a slight increase in phenolics for the two beers with added A_CE_ compared to the control sample. The total phenolics content of the beer samples with extract ranged from 226.89 to 229.11 mg GAE/L (gallic acid equivalent per liter). These values increased linearly with increasing A_CE_ concentration. The presence of phenolics in extracts of *A. oleracea* was reported by Abeysiri et al. [8], where the authors compared the phytochemical properties and antioxidant activities of different parts of this plant. The results showed significant phenolic contents in the flowers and leaves. Thus, the presence of these compounds in *Acmella* extracts indicates their use for medicinal and industrial purposes.

Ale-style beers, dark in color, with more intense alcohol content and bitterness, showed higher values of total phenolic compounds than other styles [27]. On the other hand, Lager beers, such as Pilsen and American Lager styles, are pale beers produced with low roast malts and had the lowest levels of phenolic compounds in a range of 164 to 448 mg GAE/L [28].

Despite the slight increase in TPC corresponding to 11.6% and 12.7% for B_M5_ and B_M7.5_ in relation to B_M_, the amounts of added extracts were only 0.5% (*v*/*v*) and 0.75% (*v*/*v*), respectively. The increase observed was higher than that of Humia et al. [29]. They obtained increases of 3.5 to 9.3% of TPC in beer production using Beauregard sweet potato as an adjunct to increase the antioxidant capacity. On the other hand, the increase in TPC in this study is equivalent to that found by Ulloa et al. [6] for beer added with propolis extract, in which an increase of 14.3% was observed. Ducruet et al. [5] produced beers added with Goji berries at a concentration of 5 g of fruit per liter of beer, with an increase of 20.6% in the TPC composition, a value above the B_M7.5_ sample (12.7%). Considering only the antioxidant activity, two points can be observed. First, by adding fruit to beer, other sensory and biochemical aspects are incorporated. The second point is processing, as adding fruits requires additional steps, sometimes judicious.

It is worth mentioning that the total phenolic compounds and antioxidant activities of beers depend on the style, quality, and quantity of raw material, mainly malt, and hops. The recipe was elaborated with essential ingredients (light malt and only bittering hops) to reduce the influence of the beer’s natural constituents and evaluate the effect of adding alcoholic concentrated extract (A_CE_).

### 2.4. Antioxidant Activity: ABTS, DPPH, and ORAC

Spilanthol represents about 90% of the total N-alkylamide present in *A. oleracea* [30]. The literature reports that extracts of this plant have antioxidant activities, mainly due to the presence of secondary metabolites, such as N-alkylamide, phenolics, and flavonoids [8]. HPLC analyses showed spilanthol as the main constituent of jambu extracts. Furthermore, it was observed that the jambu flower sample showed more significant free radical inhibiting activity in the DPPH radical and ABTS radical cation assays compared to extracts obtained from other parts of the plant [31]. Thygesen et al. [32] reported that, through a synergistic effect, alkamides could increase the activity of molecules with phenolic groups, consequently increasing the antioxidant capacity of the plant.

The ABTS radical cation assay showed an appreciable increase in Aa at B_M5_ and B_M7.5_. The highest radical scavenging activity was found in B_M7.5_, which showed an increase of 22.5% in relation to the control sample. The results of the DPPH radical analyses show an increase of 26.2%, with B_M7.5_ about B_M_. This percentage was higher than those published by Đorđević et al. [7]. They determined the antioxidant activity by the DPPH method in lager beers added with different extracts of medicinal plants, such as nettle root (*Urticae radix*) and lemon balm (*Melissa officinalis* L.). Increases of 12.2% (nettle) and 22.8% (lemon balm) were observed. However, in the same study, the author obtained a superior result using Thyme Herbal, where the increase was 46.5% in relation to the sample without extract.

The observed Aa values for the ABTS radical cation and DPPH radical methods agree with other studies. Tafulo et al. [33] determined the antioxidant capacity of 27 lager beers and found results from 570 to 1020 µmol ET/L for ABTS radical cation. Zhao et al. [28] published analysis results of 34 Chinese commercial beers, where values ranging from 240 to 970 µmol ET/L for DPPH radical were observed.

The results obtained by the ORAC method (Table 2) showed an important increase in antioxidant activity for B_M5_ (30.9%) and B_M7.5_ (33.7%), although these results were lower than those found by Ducruet et al. [5]. These authors produced beer enriched with goji berry at a concentration of 50 g of the fruit per liter of beer (added directly to the bottle) and obtained increases of 48% of Aa about the control sample. That study showed that goji berry is rich in bioactive compounds like polyphenols and hydroxycinnamic acids such as coumaric acid.

An important observation in this study is the reduced amount of *A. oleracea* extract. In preparing B_M5_ and B_M7.5_ beers, only 175 mg/L and 262.5 mg/L of A_CE_ were used, respectively. This did not make obtaining higher or approximate Aa values impossible compared to other authors who used higher concentrations of fruit or herbal extracts. This fact can be explained by the supercritical extraction technology, which resulted in a concentrated jambu extract.

### 2.5. Evaluation of Antioxidant Activities after Six Months of Storage

Table 2 shows the results of Aa follow-up after six months of storage. The total phenolic compounds decreased by 9.2% and 9.4% for B_M5_ and B_M7.5_, respectively. The same behavior was observed for Aa. Thus, the average decay for ABTS radical cation was 10.3%, 11.5% for DPPH radical, and 9.3% for ORAC. The Aa decay in this study was below those found by Li et al. [34], in which they analyzed the Aa of 5 commercial lager beers during six months of storage by the ABTS radical cation, DPPH, and ORAC methods, obtaining an average reduction of 42.38%, 24.44%, and 35.63% respectively. Aa decay was also studied by Martínez et al. [2] in the production of artisanal beers containing *Hibiscus sabdariffa* L. subjected to forced aging. After seven days of storage at 45 °C, there was a reduction of 31.0 and 28.1% for ABTS radical cation.

The aging of beers and, consequently, the reduction of Aa is related to the development of aging compounds, such as carbonyl, sulfur, and pyridine, which result from the oxidation of higher alcohols during storage [2,33,34]. Some studies demonstrate that beer stability is determined by its endogenous antioxidant capacity. Thus, it is interesting to increase Aa by adding extracts or brewing materials rich in bioactive compounds [35,36].

The results suggest that A_CE_ can be used to assist in the stability of Aa in beers since there was an increase in antioxidant activity. However, future studies of long-term Aa correlation by adding A_CE_ need to be carried out.

## 3. Materials and Methods

### 3.1. Material

Ground barley malt (Pilsen, Agrária, Brazil); pelleted hops (Zeus, with 15% α-acids and 4% β-acids); yeast *Saccharomyces pastorianus* (Lallemand Diamond); mineral water pH 5.5 and malt bagasse purchased from a local brewery. *A. oleracea* flowers were collected in the northern region of Brazil (Pará state), georeferenced coordinates 1°22′48.0″ S; 48°12′39.0″ W.

### 3.2. Pre-Processing of Malt Bagasse and A. olaracea Flowers

The bagasse and flowers of *A. oleracea* were dried in an air oven (Fabbe-Primar, Brazil) at 60 °C/10 h and 40 °C/24 h, respectively, until constant weight. The flowers were ground in a knife mill. Subsequently, both were stored in polypropylene plastic bags, subjected to vacuum, and stored under refrigeration until use.

### 3.3. Obtaining the Extract of A. oleracea Flowers Obtained by Supercritical Extraction (S_E_) and Preparation of the Alcoholic Concentrated Extract (A_CE_)

The *S_E_* extract was obtained using a Spe-edTM SFE (Applied Separations, model 7071, Allentown, PA, USA) coupled with a 19.7 L internal volume compressor (Schulz S/A, model CSA 78, Brazil), recirculation (Polyscience, model F08400796, Niles, IL, USA), CO_2_ (99.9% purity, White Martins, Rio de Janeiro, Brazil), co-solvent pump (1500 series) and system outlet flow meter (Alicat Scientific, Model M 5SLPM, Tucson, AZ, USA). The operational parameters used were: temperature (343 K), pressure (32 Mpa), time (30 min static and 180 min dynamic), and flow (3 L/min). The mass of dried and ground flowers was 10 g per extraction. These conditions have been described as optimal regarding spilanthol yield [14].

The extract was diluted in a minimum volume of grain alcohol for solubilization, before being added to beers. Initial dilutions were performed to obtain a homogeneous solution without dispersed particles. Thus, a concentration of 35 mg/mL (crude extract/alcohol) was established, named an alcoholic concentrated extract (A_CE_). The A_CE_ was stored under refrigeration at 8°C until the beer addition step.

### 3.4. Spilanthol Concentration in A. oleracea Extract

To confirm the presence of spilanthol in the obtained extract, separation, and analysis techniques were used.

#### 3.4.1. Thin Layer Chromatography (TLC)

Spilanthol was isolated by TLC according to the methodology proposed by Moreno et al. 2011 [37]. Briefly, 10 mg aliquots of extract obtained in the supercritical extraction were solubilized in hexane and applied to 6 nm Aluminum F-254 silica gel plates. Thus, injections of 50 µg/spot (5 µL–plate 1) of the extract and 5 µg/spot (5 µL–plate 2) of the standard (70% purity) were performed. Chromatographic plates were eluted in a glass vat using the mobile phase combination: hexane/ethyl acetate 2:1 (*v*/*v*), with a chromatographic path of 70 mm. Each plate was developed with Dragendorff’s reagent to detect the presence of alkaloids [38]. The major band corresponding to the second plate was carefully removed and again extracted with ethyl acetate. This purified fraction was used as a standard for analysis in GC-FID and FTIR–standard spilanthol (S_S_). The band corresponding to spilanthol from the first plate was analyzed by GC-FID and FTIR–extract spilanthol (S_E_).

#### 3.4.2. Gas Chromatography with Flame Ionization Detector (GC-FID)

Gas chromatography analysis was performed on a chromatograph (Thermo Fisher Scientific, Finnigan model 9001, Austin, TX, USA) equipped with a split injector (ratio 1:10). The column used was a DB-1 (J &W Scientific) fused silica capillary column (60 m × 0.32 mm × 3 μm). The oven temperature programming started at 333 K and ended at 523 K (at 3 K/min) [11]. The identification of spilanthol was made by comparison with the retention time of the isolated standard on the TLC (under the same chromatographic conditions), and quantification was performed by the area normalization method.

#### 3.4.3. Fourier Transform Infrared Analysis (FTIR)

The identification of the functional groups of the spilanthol fractions (S_E_ and S_S_) was performed by Fourier Transform Infrared Spectroscopy with Attenuated Total Reflectance (FTIR-ATR) in the range from 4000 cm^−1^ to 650 cm^−1^ (transmittance mode), resolution of 4 cm^−1^ and 32 scans. The Cary 360 (Agilent) equipment with zinc selenide (ZnSe) crystal was used.

### 3.5. Lager Beer Production

Two batches of 10 L of beer were produced: the first with 100% malt (P_M_) and the second with partial replacement by malt bagasse in the proportion of 55% (malt) and 45% (malt bagasse) coded as B_M_. The other ingredients were kept the same for both batches.

#### 3.5.1. Mash

P_M_ beer was mashed with 0.949 kg of Pilsen Malt Agrária (Brazil) and pre-heated mineral water at 70 °C at a ratio of 4:1 (*v*/*w*) (water/kg of malt). For B_M_ beer, 45% of the malt weight is replaced by malt bagasse. The temperature was maintained at 66°C for 60 min. Starch saccharification was evaluated using the iodine test (0.2 N). The mash-out was performed at 75 °C.

#### 3.5.2. Filtration and Washing

After mashing, the resulting liquid was filtered by recirculation for 25 min using the sedimented bagasse as a filtering layer and a “bazooka” type filter. After filtration, secondary water (5.3 L) at 70 °C was added on top of the filter layer to increase sugar extraction efficiency.

#### 3.5.3. Addition of Hops, Whirlpool, and Cooling

The filtered wort was boiled for 60 min. After 5 min from the start of the boil, 3.55 g of bittering hops were added. Subsequently, circular movements were performed inside the container for 5 min, followed by 20 min of rest (time for sedimentation and removal of the trub). Finally, the wort was cooled with an immersion chiller to 25 °C.

#### 3.5.4. Fermentation and Maturation

The cooled wort was transferred to the fermenter, and *Saccharomyces pastorianus* (Lallemand Diamond), previously hydrated, was inoculated in the amount prescribed by the manufacturer for 10 L. Fermentation lasted 5 days at 12 °C until the final gravity (FG) stabilized. After this step, the temperature was increased to 17 °C for 48 h to eliminate diacetyl.

Maturation took place for 5 days at 4 °C and 0.6 g dissolved gelatin/L of wort was added on the fifth day of maturation to promote beer clarification.

#### 3.5.5. Addition of the Alcoholic Concentrated Extract (A_CE_)

A_CE_ was only added to beer with malt bagasse (*B_M_*). To determine the volumes of solution to be used, preliminary sensory tests were carried out with five expert judges to establish a concentration of sensory perception that was more pleasant to the palate. These tests were carried out with commercial beer-style Pilsen with the addition of different volumes of A_CE_. Finally, 5.0 mL and 7.5 mL of A_CE_ per liter of beer were selected. The beers that received the extracts were coded as B_M5_ and B_M7.5_. A control sample was kept without the addition of A_CE_.

#### 3.5.6. Bottling and Priming

The beers (P_M_, B_M5_, and B_M7.5_) were filled in amber glass bottles with the addition of 6.5 g of crystal sugar/L of beer (priming). The bottles were stored at 24 °C for seven days. After this period, the beers were ready for consumption and analysis. A third of the bottles were separated for evaluation after six months and kept at 10 °C.

### 3.6. Physicochemical Parameters of Beers

#### 3.6.1. OG and FG

Original gravity (OG) and final gravity (FG) were measured with a densimeter and temperature correction to 20 °C. The pH values were obtained through direct reading in a digital potentiometer.

#### 3.6.2. Color

Color analysis (Official method ASBC, American Society of Brewing Chemists) [39] started with the decarbonization of the beer and membrane filtration at 0.45 nm. Subsequently, the direct reading was performed in a spectrophotometer at 430 nm. The color of the beer was evaluated according to the European Brewery Convention (EBC) from Equation (1).

Color (EBC) = A × F × 50 (1)

where, A—Is the absorbance at 430 nm in a 10 mm cuvette; F—Is the dilution factor, and 50—Is an inherent factor of the technique, for 5 mm cuvettes 25 is used.

#### 3.6.3. Bitterness (IBU)

The IBU determination was based on the content of α-acids in the beers, according to the American Society of Brewing Chemists [40]. Briefly, a 10.0 mL aliquot of decarbonated beer was added to 0.5 mL of HCl (6 mol/L) and 20 mL of isooctane. This mixture was shaken in a rotary shaker at 130 rpm for 30 min. The phase absorbance of isooctane was measured at 275 nm, and the IBU result was calculated according to equation 2.

Bitterness (IBU) = A × 50(2)

where A is the absorbance at 275 nm in a 10 mm cuvette.

#### 3.6.4. Quantifying Ethanol

Ethanol quantification was performed by high-performance liquid chromatography (HPLC); Thermo Scientific^®^ equipment (Finnigan Surveyor model), equipped with a refractive index (RI) detection system at 35 °C, using an ion exchange column (300 × 7.8 mm. Aminex^®^ HPX-87H) at 30 °C. A sulfuric acid solution (5 mM) was used as eluent with a flow rate of 0.6 mL/min for 25 min and an injection volume of 20 µL. The beer samples were previously degassed and filtered [41].

#### 3.6.5. Total Phenolic Compounds (TPC)

The content of TPC was determined by the Folin-Ciocalteau colorimetric method [42]. Briefly, a volume of 0.5 mL of diluted beer was reacted with 2.5 mL of 10% (*v*/*v*) Folin-Ciocalteu. It was allowed to react for 5 min, and then with 2 mL of 7.5% (*w*/*v*) sodium carbonate solution. The samples were kept free from light for one hour. Absorbance measurements were performed in an Evolution model UV-VIS spectrophotometer (Thermo Fisher Scientific, Waltham, MA, USA) at 760 nm. The content of phenolic compounds was expressed in milligrams of gallic acid equivalent per liter (mg GAE/L) using a calibration curve.

### 3.7. Antioxidant Activity (Aa)

#### 3.7.1. DPPH Radical Scavenging Activity

For DPPH free radical reduction, an ethanolic solution of DPPH (0.19 mM) was prepared. A volume of 2800 µL of this solution was mixed with 200 µL of sample diluted in ethanol. The absorbance was read in a spectrophotometer (Thermo Fisher Scientific, model Evolution 60, Waltham, MA, USA) at 515 nm for up to 20 min of reaction. The Trolox calibration curve was constructed as a function of DPPH• radical inhibition. Results were expressed in µmol of Trolox equivalent/L (µmol Eq Trolox/L) [43].

#### 3.7.2. ABTS Radical Cation Scavenging Activity

Initially, ABTS was diluted in water (7 mmol/L). The ABTS radical cation was produced by the reaction of the ABTS solution with 2.45 mmol/L potassium persulfate for 16 h. A volume of 2.9 mL of this solution was mixed with beer samples (0.1 mL). The absorbance was measured at 734 nm in a spectrophotometer (Thermo Fisher Scientific, model Evolution 60, Waltham, MA, USA). The Trolox calibration curve was constructed as a function of the elimination of the ABTS radical. Results were expressed in µmol of Trolox equivalent/L (µmol Eq Trolox/L) [28].

#### 3.7.3. ORAC Method

The ORAC method was performed according to Silva et al. [44]. Analysis was performed using microplates (96 wells, opaque white, Bio-One, Wemmel, Belgium) and a fluorimeter Ascent F.L. (Fluoroscan Labsystems, Helsinki, Finland)). A volume of 25 µL of the sample was mixed with 150 µL of fluorescein (55.5 nM) and incubated for 15 min at 37 °C with the subsequent addition of 25 µL of the AAPH solution (153 mM). Fluorescence was monitored for 50 min by readings at 1-min intervals (λexcitation = 485 nm; λemission= 520 nm). The calibration curve was obtained from Trolox solutions at different concentrations. All solutions were diluted in phosphate buffer (75 mM, pH 7.4). The evaluation of the antioxidant activity was based on the calculation of the area under the fluorescence decay curve. Results were expressed in µmol Trolox equivalent/L (µmol Eq Trolox/L).

All of the antioxidant activity analyses described above were repeated six months after the drinks were bottled.

### 3.8. Statistical Analysis

Statistical analysis of data from antioxidant activity tests was performed using analysis of variance (ANOVA). The comparison of the means of the experimental groups with the control was carried out using the Dunnett test, with *p* ≤ 0.05 considered significant.

## 4. Conclusions

The results clearly showed that the use of malt bagasse as a partial substitute for malt reduced the alcohol content of beer to limits that may interest a specific audience without considerably interfering with the other physicochemical properties of the beverage.

The addition of *A. oleracea* extract obtained by supercritical extraction allowed us to obtain a beer with good levels of antioxidant activity, even in minimal amounts. In addition, a global view of supercritical extraction in this study leads us to conclude that from an ecological point of view and adequacy to food safety standards, the supercritical technology satisfactorily supports obtaining concentrated extracts of *A. oleracea* that can be used in the brewing industry.

Spilanthol was the main component of the jambu flower extract. However, we cannot state that only this component was responsible for increased antioxidant activity. Other minor compounds not identified in this study may have contributed to this.

The average reduction of antioxidant activities after six months of storage was smaller than that observed by other authors when they used other raw materials. However, it will still be necessary to deepen this evaluation under different storage conditions so that the influence of these factors can be understood more broadly.

## Figures and Tables

**Table 1:** Physicochemical parameters of beers with 100% malt (P_M_), malt bagasse (B_M_), malt bagasse with 5 mL/L of A_CE_ * (B_M5_), and malt bagasse with 7.5 mL/L (B_M7.5_) of A_CE_.

Parameters	Beers (50/45% Malt/Bagasse)			
	*P_M_*	*B_M_*	*B_M5_*	*B_M_* *_7.5_*
Original Gravity (OG)	1.038 ± 0.016	1.028 ± 0.017	1.028 ± 0.016	1.028 ± 0.017
Final Gravity (OF)	1.004 ± 0.017	1.009 ± 0.017	1.009 ± 0.014	1.009 ± 0.017
pH	4.40 ± 0.002	4.10 ± 0.002	4.18 ± 0.003	4.25 ± 0.002
Color (EBC)	3.25 ± 0.4	2.20 ± 0.3	2.20 ± 0.5	2.20 ± 0.4
Bitterness (IBU)	31.29 ± 0.9	29.50 ± 0.7	29.50 ± 0.5	29.50 ± 0.8
Alcohol content (%)	4.20 ± 0.015	2.50 ± 0.016	2.50 ± 0.012	2.50 ± 0.012

* Alcoholic Concentrated Extract.

**Table 2:** TPC, ABTS, DPPH, and ORAC of control beers, with the addition of alcoholic concentrated extract (A_CE_) at different concentrations (5 and 7.5 mL/L).

Analysis	Control Beer *B_M_*	Beers Added with A_CE_ ml/L	
		*B_M5_*	*B_M7.5_*
TPC_0_ (mg GAE/L)	203.34 ± 1.52	226.89 ± 2.63 *	229.11 ± 3.62 *
TPC_6_ (mg GAE/L)	-	205.96 ± 2.55	207.57 ± 3.75
ABTS_0_ (µmol TE/L)	771.40 ± 0.01	860.30 ± 0.02 *	944.8 ± 0.02 *
ABTS_6_ (µmol TE/L)	-	781.40 ± 0.02	83,700 ± 0.09
DPPH_0_ (µmol TE/L)	265.90 ± 0.03	290.60 ± 0.05 *	335.60 ± 0.01 *
DPPH_6_ (µmol TE/L)	-	272.60 ± 0.06	317.60 ± 0.03
ORAC_0_ (µmol TE/L)	3810.84 ± 121.84	5087.21 ± 127.00 *	5396.25 ± 141.00 *
ORAC_6_ (µmol TE/L)	-	4551.11 ± 217.13	4676.67 ± 167.00

“0” (Analysis performed after the finished product) and “6” (Analysis after six months of storage). * Means differ significantly from control in the same line by Dunnett’s test (*p* > 0.05).

## Data Availability

The data presented in this study are available on request from the corresponding author.
